# Efficacy of the Nourishing Yin and Clearing Heat Therapy Based on Traditional Chinese Medicine in the Prevention and Treatment of Radiotherapy-Induced Oral Mucositis in Nasopharyngeal Carcinomas: A Systematic Review and Meta-Analysis of Thirty Randomized Controlled Trials

**DOI:** 10.1155/2022/4436361

**Published:** 2022-04-19

**Authors:** Jinsheng Huang, Jun Kan, Teng Fan, Qi Quan, Xujia Li, Qi Jiang, Bei Zhang, Guifang Guo

**Affiliations:** ^1^Guangzhou University of Chinese Medicine, Guangzhou 510006, China; ^2^VIP Department, Sun Yat-sen University Cancer Center, 651 Dongfeng Road East, Guangzhou 510006, China; ^3^State Key Laboratory of Oncology in South China, Sun Yat-sen University Cancer Center, 651 Dongfeng Road East, Guangzhou 510006, China; ^4^Collaborative Innovation Center for Cancer Medicine, Sun Yat-sen University Cancer Center, 651 Dongfeng Road East, Guangzhou 510006, China

## Abstract

This study aimed to evaluate the efficacy of nourishing Yin and clearing heat therapy (NYCH therapy) based on traditional Chinese medicine (TCM) in the treatment of radiotherapy-induced oral mucositis (RTOM) in nasopharyngeal carcinomas (NPCs). A total of eight online databases were searched from inception to September 2021 for randomized controlled trials (RCTs). The control group was treated with Western medicine (WM) alone, whereas the experimental group was treated with a combined NYCH and WM therapy. A total of 30 RCTs involving 2562 participants were ultimately included. NYCH therapy combined with conventional WM delayed the onset time (days) of RTOM (MD = 10.80, *p* < 0.001), and at that time, a higher cumulative radiotherapy dose (Gy) (MD = 5.72, *p* < 0.001) was completed in the experimental group. The combination regimen also reduced the incidence of severe oral mucositis (Grade III–IV) (RR = 0.25, *p* < 0.001). In addition, the treatment efficacy of the experimental group was significantly better than that of the control group (RR = 1.31, *p* < 0.001). Compared with the patients in the control group, the experimental group had lower xerostomia scores (MD = -1.07, *p* < 0.001) and more saliva (MD = 0.36, *p* < 0.001). NYCH combined with WM improved the efficacy of treating RTOM in NPC. This study provides a sufficient basis for conducting further large RCTs to prove the efficacy of NYCH.

## 1. Background

Nasopharyngeal carcinoma (NPC) has a distinct geographical distribution and is particularly common in East and Southeast Asia. According to the World Health Organization, the incidence of NPC in China accounts for approximately 47% of the global incidence, with the highest incidence reported in Guangdong, China [1,2]. Radiotherapy is the most important therapy for NPC, and the 5-year survival rate of patients with NPC after treatment is as high as 83.2% [3]. Despite the remarkable therapeutic effects of radiotherapy on NPC, the salivary glands are damaged after this therapy due to irradiation. The changes in the quantity, nature, and composition of saliva cause several complications, and radiotherapy-induced oral mucositis (RTOM) is the most common disease plaguing patients. More than 80% of patients with NPC suffer from RTOM during radiotherapy [4], with more than half of them developing severe RTOM (Grade III–IV) [5]. Oral mucositis is characterized by erythema and fused ulcers, and its main clinical symptoms include reduced salivation, xerostomia, oral pain, dehydration, taste disturbance, and malnutrition. In addition, severe long-term reactions can lead to difficulties in swallowing and speaking, sleep disorders, ageusia, dental caries, and oral infections [6]. Moreover, severe oral mucositis can result in reduced compliance with treatment, reduced doses of concurrent chemotherapy, or interruption of radiotherapy [7], leading to a lower quality of life, weight loss, prolonged hospital stays, and the use of additional analgesic and anti-infective drugs, thereby increasing the financial and emotional burden of patients [8]. Various therapeutic approaches have been used to prevent and treat RTOM with considerable efficacy, such as granulocyte-macrophage colony-stimulating factor (GM-CSF); recombinant human epidermal growth factor (rhEGF); borax gargle; sodium bicarbonate injection; vitamin B_12_; lidocaine gargle; analgesics such as morphine and fentanyl; antibiotics; and glucocorticoids, when necessary [9–11]. However, the efficacy of these approaches is not yet satisfactory.

In recent years, traditional Chinese medicine (TCM) practitioners have conducted several useful studies on the prevention and treatment of RTOM. The studies included Chinese medicine oral administration [12], Chinese medicine aerosol inhalation [13], Chinese medicine gargle [14], acupuncture [15], and Chinese patent medicine such as Shuangliao Houfeng Powder [16]. In Chinese medicine, oral administration is widely used, and therapies include nourishing yin and clearing heat, cooling blood and promoting fluid production, and removing toxins for relieving sore throats. Nourishing Yin and clearing heat (NYCH) decoction orally was the most common and effective in preventing and treating RTOM in NPC. However, the current available studies were all small sample sizes, and TCM efficacy in treating RTOM has not yet been elucidated in large-scale stage 3 clinical trials. RTOM is rarely treated with TCM in other countries. To assess the clinical efficacy of TCM (NYCH therapy) in treating RTOM, we conducted a literature review to retrieve clinical randomized controlled trials (RCTs) and performed a meta-analysis. In addition to the efficacy, we also investigated the medication rules of TCM in the treatment of RTOM and tried to elucidate the potential mechanism of TCM active ingredients.

## 2. Materials and Methods

### 2.1. Search Strategy

The search strategy and inclusion and exclusion criteria were developed according to the Preferred Reporting Items for Systematic Reviews and Meta-Analyses Statement (PRISMA Statement) (http://www.prisma-statement.org) (PRISMA 2020 Checklist is included in Supplement 5), followed by a literature search using a combination of electronic database and manual searches. According to the PICOS principle (population, intervention, comparators, outcomes, study design), we searched eight electronic databases, including the Excerpta Medica Database (Embase) (https://www.wolterskluwer.com/en/solutions/ovid/embase), Public Medicine (PubMed) (https://pubmed.ncbi.nlm.nih.gov), Medical Literature Analysis and Retrieval System Online (MEDLINE) (https://www.bionity.com/en/encyclopedia/MEDLINE), Cochrane Library (https://www.cochranelibrary.com), Chinese National Knowledge Infrastructure (CNKI) (https://www.cnki.net), Vipshop Chinese Journal Database (VIP) (http://www.cqvip.com), Wanfang Database (https://www.wanfangdata.com.cn), and China Biomedical Literature Service system (CBM) (http://www.sinomed.ac.cn). The search terms included ‘nasopharyngeal neoplasm, radiation therapy, oral mucositis, traditional Chinese medicine and randomized controlled trials.' The details of the search strategies are included in Supplement 1. All studies included in the search were published from the establishment of the abovementioned databases until September 2021. In addition, a manual search was conducted to avoid omissions in the literature search and to identify RCTs that might meet the inclusion criteria of the present study. All searches were restricted to human RCTs, excluding animal trials and fundamental research, and the search was conducted independently by two researchers.

### 2.2. Inclusion and Exclusion Criteria

Based on the aim of the present study, the inclusion criteria for this meta-analysis were as follows: (1) patients aged 18 years or older, male or female; (2) pathologically confirmed diagnosis of NPC, regardless of the type of pathology; (3) first concurrent radiotherapy for NPC and no previous radiotherapy to the head and neck; (4) no oral mucositis caused by other diseases; (5) RCTs, whether blinded or not; (6) complete data on outcome indicators, which can be extracted directly or indirectly for statistical analysis; (7) outcome indicators related to RTOM, (8) internal heat owing to Yin deficiency on TCM syndrome differentiation typing; and (9) full-text literature in Chinese or English that meets the aforementioned criteria.

The exclusion criteria were as follows: (1) animal studies or nonclinical studies; (2) articles with inconsistent studies or poor study designs; and (3) articles with incomplete data on outcome indicators and incorrect statistical methods that could not be corrected.

### 2.3. Interventions

The control group received conventional WM, including gentamicin, metronidazole, tinidazole, dexamethasone, prednisone, lidocaine, procaine, tetracaine, borax gargle, alpha-chymotrypsin, vitamin B12, vitamin C, rhEGF, and rhG-CSF. The detailed medicine for every study is shown in Table 1.

The experimental group received NYCH therapy combined with WM (oral administration).

### 2.4. Outcome Indicators

The outcome indicators were as follows: (1) overall effective rate of oral mucositis (the details of evaluation criteria for the overall effective rate of oral mucositis are included in Supplement 2); (2) incidence of Grade III–IV oral mucositis (the details of oral mucositis grading are included in Supplement 3.1); (3) time to the onset of oral mucositis and cumulative radiotherapy dose at the time of onset; (4) xerostomia scores (the details of xerostomia scores are included in Supplement 3.2); and (5) stimulated total saliva flow rate (the details of stimulated total saliva flow rate are included in Supplement 4).

### 2.5. Literature Screening and Data Extraction

Two trained researchers independently screened the literature, extracted data, and evaluated the methodological quality of the included RCTs according to the inclusion and exclusion criteria (Kappa index = 0.842). Disagreements were resolved through discussion or by consulting a third reviewer. A homemade form was used to extract the following data: (1) basic information about the RCTs, including the title, first author and year of publication; (2) study characteristics, including general information regarding the study object, sample size, radiotherapy dose, and interventions; and (3) the above outcome indicators.

### 2.6. Quality Assessment

The risk of bias of the included RCTs was evaluated by three trained researchers using the risk of bias assessment tool for RCTs recommended in the *Cochrane Handbook for Systematic Reviews of Interventions* [38] in RevMan (version 5.4). Disagreements were resolved via discussion or by consulting a fourth trained researcher.

### 2.7. Statistical Analysis

After collecting the data related to RCTs according to the requirements of our meta-analysis, statistical analysis was performed using RevMan (version 5.4) provided by the Cochrane Collaboration. The risk ratio (RR) was used as an effect indicator because the overall effective rate of oral mucositis and the incidence of Grade III–IV oral mucositis were dichotomous variables. However, because the remaining outcome indicators were continuous variables, the mean difference (MD) or standard mean difference (SMD) were used as effect indicators. Each effect size was expressed as a 95% confidence interval (95% CI). The chi-square (*χ*^2^) test was used to test for heterogeneity in the results of studies included in the literature. A criterion of *p* > 0.10*p* > 0.10 and I^2^ ≤ 50% indicated that there was no statistical heterogeneity among studies, and the data were combined for analysis using a fixed effects model. However, a criterion of *p* < 0.10 and I^2^ > 50% indicated statistical heterogeneity among studies and required analysis using a random effects model or, if necessary, a subgroup or sensitivity analysis. Funnel plots were used to assess potential publication bias for the included studies. For all analyses, *p* < 0.05 was considered to indicate a significant difference.

## 3. Results

### 3.1. Basic Characteristics of the Included RCTs

**Figure 1:**
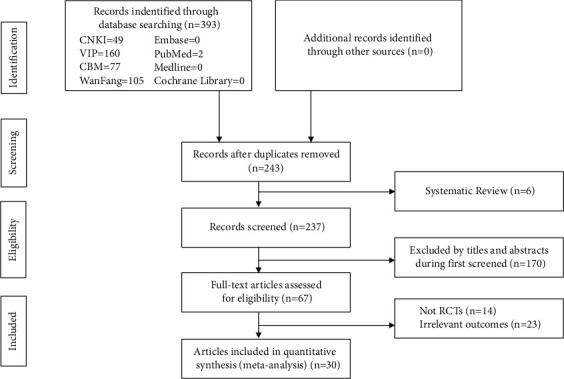
The flow diagram of the study selection process.

The literature was screened according to the PRISMA statement, and a total of 393 studies were obtained after the preliminary search. Based on the inclusion and exclusion criteria, 67 studies were selected. Eventually, 30 well-designed RCTs [12, 18–26, 28–37, 39–48] were included for meta-analysis based on full-text reading and quality assessment. The literature search process and results are shown in Figure 1.

All 30 RCTs included were conducted in China. A total of 2562 patients with pathologically confirmed NPC, aged 18–78 years, were enrolled. All patients received initial radiotherapy at a dose of 60–78 Gy and concurrent chemotherapy. The basic characteristics of the included studies are shown in Table 1.

Interventions in both the experimental and control groups were performed using conventional WM, as shown in Table 1. The patients in the experimental group received the TCM decoction orally (NYCH therapy) based on the control group. Although the formulas of the decoction varied among the experimental groups, the main prescription was to nourish Yin and clear heat.

**Figure 2:**
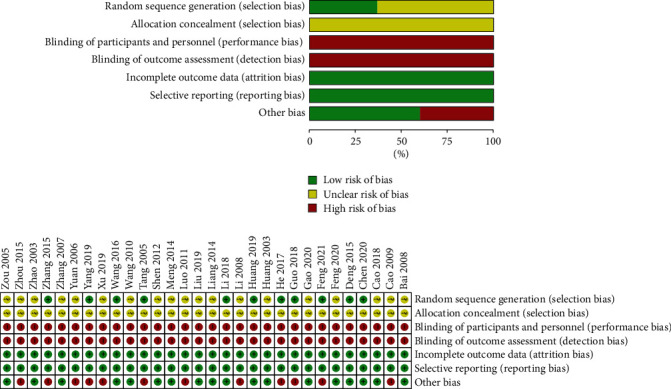
Risk of bias graph and summary. Each methodological quality item presented as percentages across all included studies and each risk of bias domain for each included study.

Baseline comparability between the experimental and control groups was confirmed by comparing baseline information on the age, sex, and condition of patients using the Cochrane Collaboration's tool for assessing the risk of bias. The complete data on outcome indicators were available for the included RCTs, and no selective reporting of study outcomes was identified. No other sources of bias were identified in the remaining literature, except for 12 RCTs [12, 23, 28, 30, 33, 34, 36, 37, 40–42, 46] with partially missing data on baseline characteristics and 1 RCT [47] (Zhou, 2015) with a small sample size (Figure 2).

### 3.2. Overall Effective Rate of Oral Mucositis

**Figure 3:**
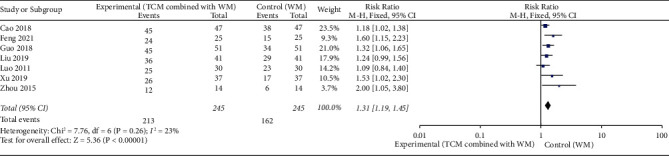
Forest plot of total effective rate, traditional Chinese medicine (TCM) combined with Western medicine (WM) showed better effect than Western medicine (WM) alone with statistical significance (RR = 1.31, *p* > 0.001).

Seven RCTs [12,18,23,28,33,44,47] (*n* = 490) reported the overall effective rate of oral mucositis, which was determined 2 weeks after radiotherapy. A healing area >1/3 of the total ulcer area or a reduction in the number of ulcers by more than 1/3 was considered effective. The meta-analysis revealed that the overall effective rate of oral mucositis was significantly better in the experimental group than in the control group (RR = 1.31, 95% CI [1.19–1.45], *p* < 0.05), with *I*^2^ = 23% (<50%) for the heterogeneity test and *p*=0.26 (>0.1) for the Q-test, suggesting slight and acceptable heterogeneity (Figure 3).

### 3.3. Incidence of Grade III–IV Oral Mucositis

**Figure 4:**
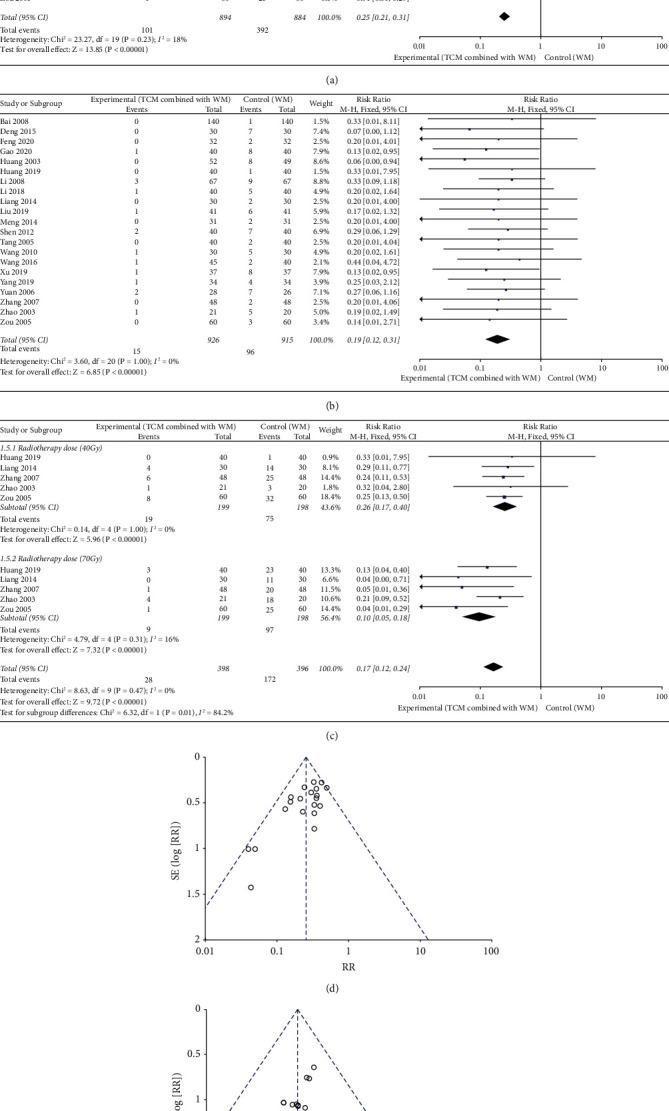
Forest plots of oral mucositis, traditional Chinese medicine (TCM) combined with Western medicine (WM) showed a better effect than Western medicine (WM) alone with statistical significance. (a) Grade III–IV oral mucositis (RR = 0.25, *p* < 0.001), (b) Grade IV oral mucositis (RR = 0.19, *p* < 0.001), and (c) Grade III–IV oral mucositis. When radiation doses reached 40 Gy (RR = 0.26, *p* < 0.001) and 70 Gy (RR = 0.10, *p* < 0.001). Funnel plots of oral mucositis, (d) Grade III–IV oral mucositis were not bilaterally symmetric, suggesting the possibility of publication bias, (e) Grade IV oral mucositis was bilaterally symmetric, suggesting no publication bias.

The meta-analysis of 21 RCTs [20–22, 24–26, 29–35, 37, 39, 42–46, 48] (*n* = 1840) revealed that the incidence of Grade III–IV oral mucositis was lower in the experimental group than in the control group (RR = 0.27, 95% CI [0.23–0.33], *p* < 0.001), with *I*^2^ = 38% (<50%) for the heterogeneity test but *p*=0.04 (<0.1) for the Q-test, suggesting that heterogeneity among the selected RCTs was significant. Therefore, to investigate the sources of heterogeneity, a sensitivity analysis was conducted on the 21 RCTs. Meng (2014) [29] was found to have a large effect on heterogeneity. After excluding this study, a meta-analysis was conducted using a fixed effects model (RR = 0.25, 95% CI [0.21–0.31], *p* < 0.001) with *I*^2^ = 18% (<50%) for the heterogeneity test and *p*=0.23 (>0.1) for the Q-test, suggesting slight and acceptable heterogeneity. Moreover, the experimental group still showed a better effect (Figure 4(a)). On the other hand, the incidence of Grade IV oral mucositis was lower in the experimental group than in the control group (RR = 0.19, 95% CI [0.12–0.31], *p* < 0.001), with *I*^2^ = 0% (<50%) for the heterogeneity test and *P* = 1.0*p*=1.0 (>0.1) for the Q-test, suggesting no heterogeneity (Figure 4(b)). After pooling the findings from the 20 RCTs, the new combined effect sizes did not change significantly when compared with the combined effect sizes before pooling, indicating low sensitivity and robust results.

Furthermore, 5 RCTs [25, 26, 35, 37, 48] (*n* = 397) that reported the incidence of Grade III–IV oral mucositis at cumulative radiotherapy doses of 40 Gy and 70 Gy were divided into two subgroups according to the cumulative radiotherapy dose for meta-analysis. The results revealed that the incidence of Grade III–IV oral mucositis in the experimental group was lower than that in the control group at a cumulative radiotherapy dose of 40 Gy (RR = 0.26, 95% CI [0.17–0.40], *p* < 0.001), with *I*^2^ = 0% (<50%) for the heterogeneity test and *p*=1.0 (>0.1) for the *Q*-test, suggesting no heterogeneity. Similarly, the incidence of Grade III–IV oral mucositis was lower in the experimental group than in the control group at the cumulative radiotherapy dose of 70 Gy (RR = 0.10, 95% CI [0.05–0.18], *p* < 0.001), with *I*^2^ = 16% (<50%) for the heterogeneity test and *p*=0.31 (>0.1) for the Q-test, suggesting slight and acceptable heterogeneity (Figure 4(c)).

Funnel plots were constructed to evaluate the publication bias among the included RCTs. No publication bias was found for Grade IV oral mucositis; however, the possibility of publication bias was found for Grade III–IV oral mucositis (Figures 4(d) and 4(e)).

### 3.4. Time to the Onset of Oral Mucositis and Cumulative Radiotherapy Dose at That Time

**Figure 5:**
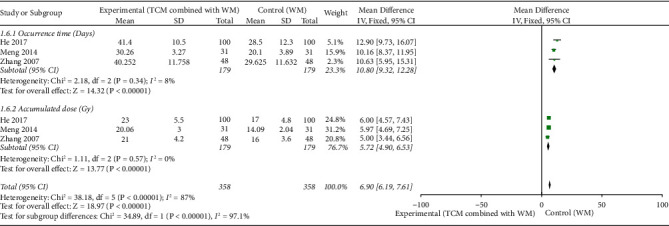
Forest plot of occurrence time (days) (MD = 10.80, *p* < 0.001) and accumulated dose (Gy) (MD = 5.72, *p* < 0.001). Traditional Chinese medicine (TCM) combined with Western medicine (WM) showed better effect than Western medicine (WM) alone with statistical significance.

Regarding the time (days) from the start of radiotherapy to the onset of Grade I oral mucositis, 3 RCTs [29, 35, 41] (*n* = 358) reported the cumulative radiotherapy dose at that moment. The meta-analysis revealed a delayed onset of Grade I oral mucositis in the experimental group compared with the control group (MD = 10.80, 95% CI [9.32–12.28], *p* < 0.001), with *I*^2^ = 8% (<50%) for the heterogeneity test and *p*=0.34 (>0.1) for the *Q*-test, suggesting slight and acceptable heterogeneity. The cumulative radiotherapy dose (Gy) at the onset of Grade I oral mucositis was higher in the experimental group than in the control group (MD = 5.72, 95% CI [4.90–6.53], *p* < 0.001), with *I*^2^ = 0% (<50%) for the heterogeneity test and *p*=0.57 (>0.1) for the *Q*-test, suggesting no heterogeneity (Figure 5).

### 3.5. Xerostomia Score

**Figure 6:**
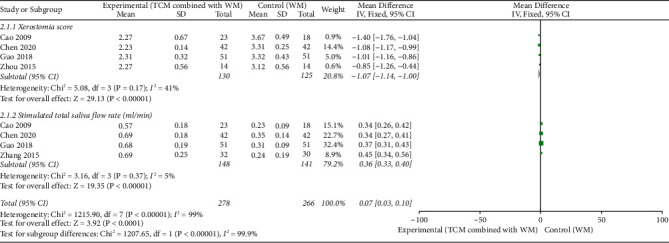
Forest plot of xerostomia score (MD = -1.07, *p* < 0.001) and stimulated total saliva flow rate (MD = 0.36, *p* < 0.001). Traditional Chinese medicine (TCM) combined with Western medicine (WM) showed better effect than Western medicine (WM) alone with statistical significance.

The meta-analysis of 4 RCTs [19, 23, 40, 47] (*n* = 255) showed that compared with the patients in the control group, patients in the experimental group had less severe xerostomia symptoms and lower xerostomia scores (MD = -1.07, 95% CI [-1.14–1.00], *p* < 0.001), with *I*^2^ = 41% (<50%) for the heterogeneity test and *p*=0.17 (>0.1) for the *Q*-test, suggesting acceptable heterogeneity (Figure 6).

### 3.6. Stimulated Total Saliva Flow Rate (mL/min)

The meta-analysis of 4 RCTs [19, 23, 36, 40] (*n* = 289) revealed that patients in the experimental group produced more saliva than patients in the control group (MD = 0.36, 95% CI [0.33–0.40], *p* < 0.001), with *I*^2^ = 5% (<50%) for the heterogeneity test and *p*=0.37 (>0.1) for the Q-test, suggesting slight and acceptable heterogeneity (Figure 6).

### 3.7. Herbal Monomers Used at High Frequencies

A total of 95 herbal monomers were used in the 30 RCTs. We found that 36 herbal monomers were used at a frequency of 3 times or more, of which 13 herbal monomers, including *Radix rehmanniae recen*, *Ophiopogon japonicus*, *Lonicera japonica*, *Radix scrophulariae*, *Adenophora stricta*, *Salvia miltiorrhiza*, Moutan bark, *Pardanthus*, *Radix Paeoniae Alba*, *Radix pseudostellariae*, *Dendrobium nobile*, *Oldenlandia diffusa*, and *Glycyrrhiza*, were used at a high frequency of 10 times or more (Table 2).

## 4. Discussion

At present, studies on the combined treatment of TCM and WM have focused on the evaluation of antitumor efficacy, whereas less attention has been devoted to adverse reactions associated with treatment. The incidence of NPC in China is the highest in the world, and oral mucositis has been the main treatment-related adverse effect affecting the survival quality of patients. In recent years, clinical studies on TCM for the treatment of RTOM in NPC have been increasingly reported in China and have shown good efficacy in patients. However, these studies had unconvincing conclusions with sample limitations and some inconsistent results. A meta-analysis was conducted in the present study by screening for clinical RCTs related to the NYCH therapy of RTOM in NPC and evaluating the efficacy of relevant outcome indicators. Our study aimed to provide higher-quality clinical evidence for TCM treatment in cases of unsatisfactory WM therapy. To date, meta-analyses related to NYCH therapy of RTOM in NPC have not been reported.

In our study, a total of 30 RCTs (*n* = 2562) were included for meta-analysis. The results revealed that NYCH therapy was effective in the prevention and treatment of RTOM in NPC. First, the overall effective rate of the experimental group was higher than that of the control group. Notably, the incidence of severe oral mucositis (Grade III–IV) was lower in the experimental group than in the control group, especially the incidence of Grade IV oral mucositis, indicating superior efficacy of the experimental group compared with the control group. Moreover, treatment efficacy was evaluated at two different points in time of a cumulative dose of 40 Gy and 70 Gy, and the results revealed that the incidence of Grade III–IV oral mucositis was significantly lower in the experimental group than in the control group at both doses. These findings suggest that NYCH therapy not only reduces the incidence of RTOM in NPC but also prevents the progression of mild or moderate-to-severe oral mucositis. Second, compared with the control group, the experimental group had a significantly delayed onset of acute oral mucositis, and acute oral mucositis only began to appear at a higher cumulative radiotherapy dose. Third, the experimental group had a significantly lower xerostomia score and a higher stimulated total saliva flow rate than the control group at the end of radiotherapy, suggesting that salivary gland secretion function was better protected during radiotherapy in the experimental group, with a lower incidence and severity of xerostomia.

According to TCM, radiation has pathogenic characteristics and is a heat-promoting and toxin-inducing procedure. Heat can be turned into fire, which can burn body fluid and exhaust Qi, resulting in Qi-Yin deficiency, thus producing symptoms such as xerostomia, sore throat, oral ulcers, and dysphagia. Therefore, TCM practitioners usually use Chinese herbal medicines that benefit Qi, nourish Yin, clear heat, and detoxify toxins to treat radiation injuries, such as RTOM.

Screening for active ingredients and assessing the underlying pharmacological mechanisms of TCM were also performed in the current research. Our study found 13 Chinese herbal medicines with a high frequency of application in NYCH therapy (Table 2). It was revealed that the main active ingredients in all 13 medicines [17, 27, 49–61] included flavonoids, terpenoids, steroids, sterols, coumarins, and emodin, which have anti-inflammatory, analgesic, and wound healing effects. The anti-inflammatory mechanism may be related to the inhibition of signaling pathways such as nuclear factor kappa-B (NF-*κ*B), mitogen-activated protein kinase (MAPK), phosphatidylinositol 3-kinase-Akt (PI3K-Akt), Janus kinase-signal transducers, and activators of transcription (JAK-STAT) [62–69]. In vitro experiments showed that acetylated iridoid glycosides obtained from *Radix scrophulariae* had a stimulating effect on the growth of human epidermal fibroblasts, which may be a potential mechanism to promote wound healing [17, 55, 56]. Future research should investigate the potential mechanisms of modern pharmacology for NYCH therapy, especially the 13 Chinese herbal medicines that were reported herein to have a high frequency of application.

The present meta-analysis had some limitations. First, most studies included had unclear methods of randomization, unclear allocation concealment, unblinded designs, no placebo controls, no loss to follow-up or withdrawals, and no intention-to-treat (ITT) analysis. Second, the experimental groups varied in drug composition, administered dose, method of administration, frequency of administration, and duration of administration. In addition, conventional WM treatments were not uniform and could not be systematically summarized.

The results of this research revealed that NYCH therapy was effective in preventing and treating RTOM in NPC, providing a basis for future multicenter and high-quality RCTs to develop guidance for clinical treatment. Modern pharmacological studies have reported herbal formulas used for NYCH therapy with a large number of anti-inflammatory and analgesic herbal monomers. These herbal medicines are viable alternatives to unsatisfactory WM therapy and may accelerate Chinese medicine pharmacology development.

## 5. Conclusion

In conclusion, a systematic review and meta-analysis in the present study demonstrated that NYCH therapy has higher efficacy in treating RTOM in NPC, with a higher overall effective rate, lower incidence of RTOM, and delayed onset, preventing the progression of mild or moderate-to-severe oral mucositis and relieving serious xerostomia.

## Figures and Tables

**Table 1:** The basic characteristics of all included randomized controlled trials' studies.

Study	No	Age	Male	Female	Intervention	Radiation dose evaluation	
	(E/C)	(E/C)	(E/C)	(E/C)	(E/C)		
[17]	140/140	mean = 45.3Y/ mean = 44.9Y	86/88	54/52	NYCH therapy + D1, D4, D8, D11, D12, D15/D1, D4, D8, D11, D12, D15	72 to 78 Gy	OMR (G III–IV)
[18]	23/18	24 to 68 (mean = 52.2) Y	27^*∗*^	14^*∗*^	NYCH therapy + D10/D10	60 to 76 Gy	TER, XS,
SFR							
[18]	47/47	26 to 69 (mean = 51.05 ± 6.47) Y/29 to 68 (50.61 ± 6.89) Y	33/32	14/15	NYCH therapy + D1, D4, D11/D1, D4, D11	60 to 75 Gy	TER
[19]	42/42	43 to 71 (mean = 56.54 ± 6.37) Y/45 to 73 (mean = 57.89 ± 6.19) Y	29/27	13/15	NYCH therapy + D11/D11	68 to 76 Gy	XS, SFR
[20]	30/30	35 to 72 (mean = 56.7 ± 8.9) Y/38 to 75 (mean = 58.3 ± 10.4) Y	17/20	13/10	NYCH therapy + D14/D14	68 to 74 Gy	OMR (G III–IV)
[21]	32/32	mean = 52.91 ± 3,.82Y/mean = 53.57 ± 4.25Y	19/18	13/14	NYCH therapy + D1, D4, D11/D1, D4, D11	NA	OMR (G III–IV)
[12]	25/25	mean = 49.6 ± 5.6Y/ mean = 50.1 ± 6.0Y	15/13	10/12	NYCH therapy + D15/D15	NA	TER
[22]	40/40	30 to 72 (mean = 55.15 ± 6.01) Y/30 to 73(mean = 54.61 ± 5.92) Y	28/26	12/14	NYCH therapy + D14/D14	66 to 72 Gy	OMR (G III–IV)
[23]	51/51	mean = 52 ± 1.27Y/ mean = 54 ± 2.01Y	30/32	21/19	NYCH therapy + D17/D17	64 to 70.4 Gy	TER, XS,
SFR							
He et al. 2017	100/100	21 to 71Y/23 to 69Y	72/74	28/26	NYCH therapy + D2/D2	NA	OMR (TC)
[24]	52/49	20 to 70 (mean = 45) Y/22 to 71(mean = 46) Y	42/40	10/9	NYCH therapy + D14/D14	68 to 72 Gy	OMR (G III–IV)
[25]	40/40	mean = 47.76 ± 5.37Y/mean = 48.23 ± 5.72Y	27/25	13/15	NYCH therapy + D15/D15	68 to 74 Gy	OMR (G III–IV)
Li et al. 2008	67/67	mean = 46.6Y/ mean = 46.1Y	57/51	10/16	NYCH therapy + D14/D14	62 to 74 Gy	OMR (G III–IV)
Li et al. 2018	40/40	mean = 44.48 ± 5.23Y/mean = 44.75 ± 4.72Y	29/26	11/14	NYCH therapy + D1, D6, D9/D1, D6, D9	70 Gy	TER, OMR (G III–IV)
[26]	30/30	24 to 78 (mean = 43) Y	42^∗^	18^∗^	NYCH therapy + D1, D5, D9, D14/D1, D5, D9, D14	68 to 72 Gy	OMR (G III–IV)
[27]	41/41	mean = 52.80 ± 7.26Y/mean = 52.43 ± 7.18Y	27/25	14/16	NYCH therapy + D16/D16	66 to 72 Gy	TER, OMR (G III–IV)
[28]	30/30	19 to 66 (mean = 46.33 ± 11.85) Y/18 to 69(mean = 44.90 ± 13.32) Y	21/18	9/12	NYCH therapy + D17/D17	64 to 70 Gy	TER
[29]	31/31	mean = 43.20 ± 7.79Y/ mean = 43.4 ± 8.86Y	22/24	9/7	NYCH therapy + D1, D4, D6, D9/D1, D4, D6, D9	66 to 70 Gy	OMR (G III–IV), OMR (TC)
Shen et al. 2012	40/40	mean = 50.22 ± 10.17Y/mean = 50.65 ± 11.25Y	31/29	9/11	NYCH therapy + D1, D4, D6/D1, D4, D6	70 to 74 Gy	OMR (G III–IV)
[30]	40/40	21 to 68 (mean = 47.5) Y/23 to 70(mean = 48.5) Y	NA	NA	NYCH therapy + D17/D17	60 to 70 Gy	OMR (G III–IV)
[31]	30/30	mean = 46.3 ± 11.5Y/mean = 45.3 ± 13.0Y	25/24	5/6	NYCH therapy + D2/D2	60 to 70 Gy	OMR (G III–IV)
[32]	45/40	mean = 50.34 ± 12.06Y/mean = 52.83 ± 8.37Y	24/25	21/15	NYCH therapy + D1, D4, D7/D1, D4, D7	68 to 76 Gy	OMR (G III–IV)
[33]	37/37	24 to 71 (mean = 45.41 ± 1.50) Y/23 to 71 (mean = 45.32 ± 1.51) Y	25/22	12/15	NYCH therapy + D17/D17	60 to 75 Gy	TER, OMR (G III–IV)
[34]	34/34	45∼72 (57.26 ± 9.71)/46∼70(57.05 ± 8.82)	20/21	14/13	NYCH therapy + D11/D11	NA	OMR (G III–IV)
Yuan et al. 2006	28/26	30 to 70 (mean = 48.5) Y/25 to 72(mean = 46.8) Y	15/14	13/12	NYCH therapy + D17/D17	60 to 70 Gy	OMR (G III–IV)
[35]	48/48	21 to 72 (mean = 45) Y/20 to 72(mean = 46) Y	40/40	8/8	NYCH therapy + D1, D4, D6, D9, D13/D1, D4, D6, D9, D13	68 to 76 Gy	OMR (G III–IV), OMR (TC)
[36]	32/30	30 to 64 (mean = 48.4) Y/29 to 64(mean = 49.3) Y	18/18	14/12	NYCH therapy + D17/D17	70 to 76 Gy	SFR
[37]	21/20	30 to 72(mean = 46) Y/27 to 69(mean = 50) Y	13/11	8/9	NYCH therapy + D17/D17	68 to 70 Gy	OMR (G III–IV)
Zhou et al. 2015	14/14	32 to 63(mean = 47.5) Y/38 to 66(mean = 48.1) Y	8/7	6/7	NYCH therapy + D10/D10	60 to 76 Gy	TER, XS
Zou et al. 2005	60/60	18 to 72(mean = 42) Y/20 to 73(mean = 43) Y	55/54	5/6	NYCH therapy + D14/D14	68 to 72 Gy	OMR (G III–IV)

*Note.* NYCH therapy = nourishing Yin and clearing heat therapy; E/C = experimental groups/control groups; Y = year(s); ^*∗*^male and female not grouped; NA= not applicable; D = drug; D1 = gentamicin; D2 = metronidazole; D3 = tinidazole; D4 = dexamethasone; D5 = prednisone; D6 = lidocaine; D7 = procaine; D8 = tetracaine; D9 = vitamin B12; D10 = vitamin C; D11 = Kangfuxin solution; D12 = recombinant human epidermal growth factor, rhEGF; D13 = chymotrypsin; D14 = compound borax solution; D15 = compound chlorhexidine gargle; D16 = recombinant human granulocyte colony-stimulating factor injection, rhG-CSF; D17 = conventional Western medicine; TER = total effective rate; OMR (G III–IV) = Grade III–IV oral mucositis; OMR (TC) = time and cumulative of oral mucositis; XS = xerostomia score; SFR=saliva dynamic total flow rate.

**Table 2:** High frequency monomer of TCM used 10 times or more.

Herbs	RCT
*Radix rehmanniae recen*	29
*Ophiopogon japonicus*	27
*Glycyrrhiza*	24
*Lonicera japonica*	16
*Radix scrophulariae*	16
*Adenophora stricta*	14
Moutan bark	11
*Radix pseudostellariae*	11
*Salvia miltiorrhiza*	11
*Pardanthus*	10
*Dendrobium nobile*	10
*Radix Paeoniae Alba*	10
*Oldenlandia diffusa*	10

## Data Availability

All the data generated or analyzed during this study are included within the article.
